# Obese adolescents have higher risk for femur fracture after motor vehicle collision

**DOI:** 10.1016/j.sopen.2024.07.007

**Published:** 2024-07-22

**Authors:** Shaelyn Choi, Jeffry Nahmias, Matthew Dolich, Michael Lekawa, Brian R. Smith, Ninh Nguyen, Areg Grigorian

**Affiliations:** University of California, Irvine, Department of Surgery, Division of Trauma, Burns and Surgical Critical Care, Orange, CA, USA

**Keywords:** Adolescent, Obesity, Femur fracture, Motor vehicle collision

## Abstract

**Background:**

Previous reports identified an association between obese adolescents (OAs) and lower extremity (LE) fractures after blunt trauma. However, the type of LE fracture remains unclear. We hypothesized that OAs presenting after motor vehicle collision (MVC) have a higher risk of severe LE fracture and will require a longer length of stay (LOS) and more support services upon discharge, compared to non-OAs.

**Methods:**

The 2017–2019 Trauma Quality Improvement Program database was queried for adolescents (12–17-years-old) presenting after MVC. The primary outcome was LE fracture. A severe fracture was defined by abbreviated injury scale ≥3. OAs were defined by a body mass index (BMI) ≥30.

**Results:**

From 22,610 MVCs, 3325 (14.7 %) included OAs. The rate of any LE fracture was higher for OAs (21.6 % vs. 18.8 %, *p* < 0.001). On subset analysis the only LE fracture at higher risk in OAs was a femur fracture (13 % vs. 9.1 %, *p* < 0.001). After adjusting for sex and age, the risk for severe LE fracture (OR 1.34, CI 1.18–1.53, *p* < 0.001) was higher for OAs. OAs with a femur fracture had a longer median LOS (5 vs. 4 days, *p* = 0.003) and were more likely discharged with additional support services including home-health or inpatient rehabilitation (30.6 % vs. 21.4 %, *p* < 0.001).

**Conclusion:**

OAs sustaining MVCs have increased associated risk of femur fractures. OAs are more likely to have a higher-grade LE injury, experience a longer LOS, and require additional support services upon discharge. Future research is needed to determine if early disposition planning with social work assistance can help shorten LOS.

## Introduction

Obesity in adolescence is a health condition that has become endemic [1]. The World Health Organization reports that in the past three decades, the prevalence of obese and overweight adolescents has increased from 4 % to nearly 20 % globally [1]. The Centers for Disease Control and Prevention state that nearly 20 % of adolescents in the United States are obese, affecting over 14 million children and adolescents [2]. Obesity has been associated with multiple comorbidities including insulin resistant diabetes, hypertension, hyperlipidemia, and an increased risk of musculoskeletal injuries [[3], [4], [5], [6]].

Obesity may have a more profound impact during adolescence compared to adulthood. In the peak period of adolescent growth, there is a higher incidence of fractures due to a mismatch in bone turnover rates [[7], [8], [9]]. For obese adolescents (OAs), the detrimental effect of excess fat mass may exacerbate this issue [[7], [8], [9], [10]]. Although it has been suggested that OAs may have increased bone size and bone density compared to non-obese adolescents (non-OAs), this may not be true after adjusting for body weight, potentially increasing their risk of fracture after trauma [[11], [12], [13]].

The impact of obesity on the incidence of extremity fractures after trauma in OAs has mixed reports, and even less is known about its impact on adolescent trauma patients presenting after a motor vehicle collision (MVC), one of the most common mechanisms of trauma in the United States [14]. Obesity may lead to more severe injuries, a higher risk of complications, and increased mortality after trauma [15]. Some obese trauma patients may require additional support services upon discharge including physical rehabilitation. Previous studies have shown an increased risk of lower extremity fractures after MVC in obese patients [[16], [17], [18]]. However, no studies have investigated the severity of lower extremity fractures in OAs after MVC, and the rate at which these patients may require support services upon discharge remains unknown.

This study aims to identify the impact of obesity on extremity fractures sustained by adolescents during MVCs, hypothesizing that OAs have a higher risk of severe lower extremity fractures compared to non-OAs. Additionally, we hypothesize that OAs with lower extremity fractures have a longer length of stay (LOS) and a higher incidence of requiring support services upon discharge.

## Materials and methods

This study was deemed exempt by our local Institutional Review Board and written informed consent was waived as it uses a national de-identified database. The 2017–2019 Trauma Quality Improvement Program (TQIP) database was queried for patients aged 12 to 17 years presenting after MVC. Body mass index (BMI) was calculated using weight (kg) and height. Two groups were compared: OA with a BMI ≥ 30 kg/m^2^ and non-OA with a BMI < 30 kg/m^2^. Outlier patients (BMI < 13 and BMI > 60 kg/m^2^) and patients without a complete set of height and weight were excluded from the analysis. The primary outcome was lower extremity fracture comprising of femur fracture, knee fracture, tibia fracture, fibula fracture, ankle fracture, and foot fracture. Severe lower extremity fracture was defined by an abbreviated injury score (AIS) ≥ 3. The AIS is a standardized scoring system used to classify and describe the severity of injuries. Scores range from 1 (minor) to 6 (maximal, currently untreatable), with a score of ≥3 indicating a severe injury. This definition allows for a consistent and objective assessment of fracture severity, facilitating comparison with other studies.

Demographic variables collected include age, sex, and comorbidities, which include attention deficit hyperactivity disorder (ADHD), congenital abnormality, diabetes, hypertension, and mental personality disorder. The injury profile included fractures to the rib, upper extremity, and pelvis, and injuries to the brain, cardiac, lung, small intestine, colon, spleen, liver, pancreas, and kidney. Vital signs upon admission, injury severity score (ISS) and Glasgow Coma Scale (GCS) were also reported.

We also collected data on in-hospital complications including cardiac arrest, catheter-associated urinary tract infection (CAUTI), central line-associated bloodstream infection (CLABSI), deep surgical site infection, deep vein thrombosis, pulmonary embolism, unplanned intubation, acute kidney injury, myocardial infarction, pressure ulcer, acute respiratory distress syndrome, unplanned return to operating room (OR), sepsis, stroke, superficial surgical site infection, unplanned ICU admission, ventilator-associated pneumonia, and death. Other clinical outcomes include hospital LOS, intensive care unit (ICU) LOS and ventilator days. A subset analysis of patients with femur fractures was also performed.

For all variables, descriptive statistics were performed. Mann-Whitney-*U* test was used to compare continuous variables and chi-square test was used to compare categorical variables. Continuous variables were reported as medians with an interquartile range (IQR) and categorical variables were reported as percentages. A multivariable logistic regression model was then used to determine the risk of lower extremity fracture after MVC controlling for age and sex as these variables are related with differences in bone mineral density, bone size, and bone accrual which may influence the rates of lower extremity fractures [[19], [20], [21]]. The adjusted risk for lower extremity fractures was reported with an odds ratio (OR) and 95 % confidence intervals (CI). We performed additional subset analyses for various ranges of BMI (30–34.99, 35–39.99, and ≥ 40). The reference range for these analyses included patients with a normal BMI range of 18.5–24.9. All *p*-values were two-sided with a statistical significance level of <0.05. All analyses were performed with IBM SPSS Statistics for Windows (Version 28, IBM Corp., Armonk, NY).

## Results

### Demographics and injury profiles of OAs and non-OAs involved in MVCs

From 22,610 MVCs, 3325 (14.7 %) involved OAs. The median BMI was 33.9 kg/m^2^ for OAs and 21.8 kg/m^2^ for non-OAs (*p* < 0.001). There were no differences in age and sex between the two groups (all *p* > 0.05). Compared to non-OAs, OAs had a higher prevalence of diabetes (1.4 % vs. 0.5 %, *p* < 0.001), hypertension (0.9 % vs. 0.2 %, p < 0.001), and mental personality disorder (4.2 % vs. 3.1 %, *p* < 0.001) (Table 1). OAs presented with a higher rate of any lower extremity fracture (21.6 % vs. 18.8 %, *p* < 0.001) and severe lower extremity fracture (17.7 % vs. 13.9 %, p < 0.001). The only fracture with a higher incidence in OAs was femur fracture (13.0 % vs. 9.1 %, p < 0.001); rates of other lower extremity fractures (knee, fibula, ankle, and foot fractures) were similar between groups (all *p* > 0.05) For solid organ injuries, OAs had lower rates of lung (16.2 % vs. 21.6 %, *p* < 0.001), small intestine (1.0 % vs. 1.5 %, *p* = 0.017), colon (0.8 % vs. 1.3 %, *p* = 0.041), spleen (6.0 % vs. 7.0 % *p* = 0.048), and liver (5.2 % vs. 6.1 %, *p* = 0.040) injuries compared to non-OAs **(**Table 2**).**

**Table 1:** Demographics of OA vs non-OA patients involved in motor vehicle collisions.

Demographics	Non-OA	OA	*p*-value
	(*n* = 19,285)	(*n* = 3325)	
BMI, median (IQR)	21.8 (5)	33.9 (6)	<0.001
BMI, categorized, n (%)			
30–34.99		1937 (58.3 %)	
35–39.99		817 (24.6 %)	
≥40		571 (17.2 %)	
Age, year, median (IQR)	15 (2)	15 (2)	0.694
Male, n (%)	11,224 (58.2 %)	1918 (57.7 %)	0.573
Comorbidities, n (%)			
ADHD	1166 (6.1 %)	206 (6.2 %)	0.734
Congenital abnormality	172 (0.9 %)	31 (0.9 %)	0.819
Diabetes	102 (0.5 %)	45 (1.4 %)	<0.001
Hypertension	33 (0.2 %)	31 (0.9 %)	<0.001
Mental Personality Disorder	589 (3.1 %)	139 (4.2 %)	<0.001

OA = Obese Adolescents; BMI = Body Mass Index; IQR = Interquartile Range; ADHD = Attention Deficit Hyperactivity Disorder.

**Table 2:** Vital signs and injury profiles of OAs vs non-OAs involved in motor vehicle collisions.

Vital Signs and Injury	Non-OA	OA	
	(n = 19,285)	(n = 3325)	p-value
Vital Signs, n (%)			
Hypotensive (<90 mmHg)	347 (1.8 %)	65 (2.0 %)	0.513
Tachycardia (>120/min)	2143 (11.2 %)	533 (16.2 %)	<0.001
Tachypnea (>22/min)	4348 (23.1 %)	830 (25.6 %)	0.002
Injury Severity Score > 15	3965 (20.6 %)	622 (18.8 %)	0.014
Glasgow Coma Scale ≤8	1449 (7.7 %)	223 (6.9 %)	0.104
Injury, n (%)			
Traumatic Brain Injury	3215 (16.7 %)	496 (14.9 %)	0.012
Cardiac	90 (0.5 %)	16 (0.5 %)	0.910
Rib fracture	1839 (9.5 %)	3325 (14.7 %)	0.018
Lung	4156 (21.6 %)	539 (16.2 %)	<0.001
Small Intestine	295 (1.5 %)	33 (1.0 %)	0.017
Colon	243 (1.3 %)	28 (0.8 %)	0.041
Spleen	1347 (7.0 %)	201 (6.0 %)	0.048
Liver	1180 (6.1 %)	173 (5.2 %)	0.040
Pancreas	107 (0.6 %)	11 (0.3 %)	0.098
Kidney	628 (3.3 %)	123 (3.7 %)	0.188
Pelvis Fracture	1815 (9.4 %)	339 (10.2 %)	0.155
Upper Extremity Fracture	3031 (13.4 %)	591 (17.8 %)	0.003
Lower Extremity Fracture, n (%)	3627 (18.8 %)	717 (21.6 %)	<0.001
Femur Fracture	1764 (9.1 %)	432 (13.0 %)	<0.001
Knee Fracture	111 (0.6 %)	19 (0.6 %)	0.977
Tibia Fracture	1239 (6.4 %)	175 (5.3 %)	0.011
Fibula Fracture	854 (4.4 %)	155 (4.7 %)	0.547
Ankle Fracture	476 (2.5 %)	101 (3.0 %)	0.055
Foot Fracture	634 (3.3 %)	117 (3.5 %)	0.492
Severe⁎ Lower Extremity fracture, n (%)	2676 (13.9 %)	587 (17.7 %)	<0.001

OA = Obese Adolescents

⁎ *AIS = Abbreviated Injury Score ≥ 3.*

### Outcomes of OAs vs non-OAs involved in MVCs with femur fracture

Given that femur fractures were the only type of lower extremity fracture occurring more frequently in OAs, we performed a subset analysis in patients with a femur fracture. The mortality rate was similar (*p* = 0.37) for both groups (Table 3**)**. However, OAs with a femur fracture had a longer median LOS compared to non-OAs with a femur fracture (5 vs. 4 days, *p* = 0.003). OAs experienced higher rates of pulmonary embolism (1.4 % vs. 0.2 %, *p* < 0.001) and sepsis (0.5 % vs. 0.1 %, *p* = 0.040). Additionally, OAs with femur fracture were discharged with support services more frequently than non-OAs (30.6 % vs. 21.4 %, p < 0.001) (Table 4).

**Table 3:** Clinical outcomes of OAs vs non-OAs involved in motor vehicle collisions.

Outcomes	Non-Obese (*n* = 19,285)	Obese (*n* = 3325)	p-value
Length of Stay, days, median (IQR)	3 (3)	3 (3)	0.732
ICU Length of Stay, days, median, (IQR)	3 (3)	3 (3)	0.709
Ventilation, days, median, (IQR)	3 (4)	3 (5)	0.577
Complications, n (%)	396 (2.1 %)	90 (2.7 %)	0.016
Cardiac Arrest	48 (0.2 %)	10 (0.3 %)	0.580
CAUTI	28 (0.1 %)	8 (0.2 %)	0.201
CLABSI	6 (0.0 %)	2 (0.1 %)	0.410
Deep Surgical Site Infection	15 (0.1 %)	3 (0.1 %)	0.812
Deep Vein Thrombosis	50 (0.3 %)	16 (0.5 %)	0.028
Pulmonary Embolism	8 (0.0 %)	10 (0.3 %)	<0.001
Unplanned Intubation	35 (0.2 %)	14 (0.4 %)	0.006
Acute Kidney Injury	31 (0.2 %)	9 (0.3 %)	0.162
Pressure Ulcer	53 (0.3 %)	18 (0.5 %)	0.011
Acute Respiratory Distress Syndrome	39 (0.2 %)	10 (0.3 %)	0.257
Unplanned Return to OR	62 (0.4 %)	15 (0.5 %)	0.232
Sepsis	11 (0.1 %)	4 (0.1 %)	0.189
Stroke	15 (0.1 %)	4 (0.1 %)	0.432
Superficial Surgical Site Infection	18 (0.1 %)	6 (0.2 %)	0.153
Unplanned ICU Admission	76 (0.4 %)	25 (0.8 %)	0.004
Ventilator-Associated Pneumonia	75 (0.4 %)	15 (0.5 %)	0.593
Death, n (%)	241 (1.2 %)	42 (1.3 %)	0.949

OA = Obese Adolescents; IQR = Interquartile Range; ICU = Intensive Care Unit; CAUTI = Catheter-Associated Urinary Tract Infection; CLABSI = Central Line-Associated Bloodstream Infection; OR = Operating Room.

**Table 4:** Clinical outcomes in OA vs non-OA with femur fracture involved in motor vehicle collisions.

Outcomes	Non-Obese (*n* = 1750)	Obese (*n* = 428)	p-value
Length of Stay, days, median (IQR)	4 (5)	5 (6)	0.003
ICU Length of Stay, days, median, (IQR)	4 (6)	4 (7)	0.428
Ventilation, days, median, (IQR)	3 (5)	5 (8)	0.092
Discharged with help, n (%)	378 (21.4 %)	132 (30.6 %)	<0.001
Complications, n (%)	73 (4.1 %)	26 (6.0 %)	0.091
Cardiac Arrest	10 (0.6 %)	4 (0.9 %)	0.400
CAUTI	3 (0.2 %)	2 (0.5 %)	0.252
CLABSI	3 (0.2 %)	0 (0.0 %)	0.391
Deep Surgical Site Infection	1 (0.1 %)	0 (0.0 %)	0.621
Deep Vein Thrombosis	15 (0.9 %)	4 (0.9 %)	0.879
Pulmonary Embolism	3 (0.2 %)	6 (1.4 %)	<0.001
Unplanned Intubation	5 (0.3 %)	2 (0.5 %)	0.553
Acute Kidney Injury	5 (0.3 %)	2 (0.5 %)	0.553
Pressure Ulcer	11 (0.6 %)	5 (1.2 %)	0.242
Acute Respiratory Distress Syndrome	11 (0.6 %)	3 (0.7 %)	0.868
Unplanned Return to OR	8 (0.5 %)	2 (0.1 %)	0.981
Sepsis	1 (0.1 %)	2 (0.5 %)	0.040
Stroke	7 (0.4 %)	0 (0.0 %)	0.190
Superficial Surgical Site Infection	2 (0.1 %)	2 (0.5 %)	0.127
Unplanned ICU Admission	12 (0.7 %)	7 (1.6 %)	0.059
Ventilator-Associated Pneumonia	11 (0.6 %)	3 (0.7 %)	0.868
Death, n (%)	41 (2.3 %)	7 (1.6 %)	0.370

OA = Obese Adolescents; IQR = Interquartile Range; ICU = Intensive Care Unit; CAUTI = Catheter-Associated Urinary Tract Infection; CLABSI = Central Line-Associated Bloodstream Infection; OR = Operating Room.

### Multivariable logistics regression analyses controlled for age and sex

After adjusting for age and sex, OAs had a higher risk of lower extremity fractures (OR 1.20, CI 1.06–1.35, *p* = 0.003) and severe lower extremity fractures (OR 1.34, CI 1.18–1.53, *p* < 0.001) compared to non-OAs. When analyzing lower extremity fractures by location, only femur fractures was shown to occur with a higher risk in OAs compared to non-OAs (OR 1.47, CI 1.27–1.71, p < 0.001). There was a gradation of risk of lower extremity fractures, severe lower extremity fractures, and femur fractures with higher BMI ranges (*p* < 0.05) (Table 5**)**.

**Table 5:** Multivariable⁎ logistics regression analysis⁎⁎ for risk of lower extremity fracture, severe lower extremity fracture, and femur fracture in MVCs.

Risk Factor	OR	CI	p-value
Lower Extremity Fracture			
BMI 30–34.99	1.20	1.06–1.35	0.003
BMI 35–39.99	1.22	1.09–1.39	0.004
BMI ≥ 40	1.40	1.15–1.71	<0.001
Severe+ Lower Extremity Fracture			
BMI 30–34.99	1.32	1.09–1.60	0.004
BMI 35–39.99	1.34	1.18–1.53	<0.001
BMI ≥ 40	1.47	1.18–1.82	<0.001
Femur Fracture			
BMI 30–34.99	1.47	1.27–1.71	<0.001
BMI 35–39.99	1.59	1.29–1.97	<0.001
BMI ≥ 40	1.80	1.41–2.29	<0.001

OA = Obese Adolescents

⁎ *= controlled for sex and age*

⁎⁎ *= reference range includes normal BMI (18.5–24.9)*

+ *= AIS (Abbreviated Injury Score) ≥ 3.*

## Discussion

MVCs are a leading cause of injury in the United States [14]. As the obesity epidemic continues to grow, the rate at which we see obese trauma patients presenting after MVC will only continue to rise [22]. This retrospective study examines the impact of obesity on lower extremity fractures sustained in MVCs and finds that OAs have a higher risk of severe femur fractures. The risk is positively correlated with increased BMI ranges. OAs with femur fractures have a longer LOS and are discharged with more support services compared to non-OAs. OAs exhibit a lower rate of solid organ injuries than non-OAs.

While obesity is associated with a higher risk of lower extremity fractures, previous research has not analyzed different types or severity of fractures, leading to conflicting evidence on injury patterns among OAs [6,23]. Zaveri et al. found that there was no association between injuries to specific body regions and BMI [24]. In contrast, a systematic review and meta-analysis of adult trauma patients involved in MVC found obesity to be associated with a higher risk of lower extremity fractures [25]. Our study partially confirms this to be true in OAs as we only found the femur to be associated with a higher risk of fracture. There are several reasons why the femur may be the only bone at risk for fracture after MVC in OAs. The femur is often directly impacted during an MVC and absorbs a large amount of the total force which may result in fracture [16,26]. Additionally, a prior report found that obese cadavers experienced greater hip excursion during a biomechanical experimental study which subsequently caused more lower extremity movement resulting in an increased risk of hard contact with surrounding barriers [27]. This may explain why OAs have a higher risk of severe lower extremity fracture.

Although obese patients generally have higher bone mineral density, this increase is insufficient to withstand the increased loads, making them more susceptible to fractures [12,28,29]. This susceptibility may be exacerbated during puberty and periods of rapid growth due to hormonal influences and excess leptin, which can negatively affect bone turnover [8,9]. Interestingly, *Cao* et al. found that mice with a high fat diet had a lower femoral bone mass compared to increased lumbar bone mass which suggests that bone health and the impact of obesity may be subject to location [30]. Negative alterations in femur bone microarchitecture especially during puberty, in combination with the high forces sustained to the femur during MVCs, may explain why we found the femur to be the only bone at higher risk of fracture for OAs.

Obesity has been linked to a longer LOS in both pediatric and adult trauma patients [[31], [32], [33], [34]]. Our study supports this finding, with OAs having a longer LOS and requiring more support services upon discharge than non-OAs. Obesity has been known to be a risk factor for an increased incidence of pulmonary embolism [35,36]. Our study showed that OAs had a higher rate of PE which may be one potential reason for the longer LOS. In adult populations, obesity has been associated with a greater need for support services upon discharge [37,38]. We confirm this in OAs with femur fracture. Delayed mobility may increase risk for complications and LOS indicating that early, comprehensive mobilization protocols may help improve OA patient's LOS and lessen the need for discharge with rehabilitation services [33]. Future research is needed to determine if LOS can be shortened with early disposition planning with social work assistance or with more aggressive mobilization strategies, if reasonable. In addition, planning comprehensive discharge services to include inpatient and outpatient rehabilitation services should be emphasized.

Obesity may serve as a “protective” barrier between a traumatic force and injury. The “Cushion Effect” is a potential explanation as to why obese patients may experience different injury patterns and fewer abdominal injuries due to an increased amount of subcutaneous fat [[39], [40], [41]]. There is conflicting evidence regarding the existence of the “Cushion Effect”. *Tee* et al. found that increased subcutaneous fat was not associated with abdominal injury in children and adolescents while other single-center studies found subcutaneous fat to be protective. Interestingly, *Arbabi* et al. found that overweight but not obese patients were associated with higher risk of abdominal trauma, compared to normal weight patients [42]. Increased subcutaneous fat is often correlated with BMI, and thus the increased subcutaneous fat can act to absorb the energy experienced during an MVC, providing a protective effect against abdominal injuries [41,43]. Although our intent was to study the risk of lower extremity fracture, this study adds to the existing body of literature as we found a lower rate of solid organ injury (liver and spleen) in OAs presenting after MVC [39]. The lower rate of solid organ injury and higher rate of lower extremity fractures in OAs suggests that OAs may have different injury profiles than their non-OA counterparts. Awareness of the unique injury profiles may help improve clinical outcomes and the efficiency of healthcare for OAs presenting after MVC.

Limitations of this study include those inherent to the use of the large TQIP database including selection bias, missing data, and misclassification errors. The TQIP database does not include more granular information regarding the nature of the MVC or the location of patients in the vehicle to account for potential confounding factors. TQIP does not capture the detailed biomechanical data from the MVCs, such as the specific forces involved, the direction of impact, and the use of safety features like seat belts and airbags. Another limitation of the database is that it does not account for the types of femur fractures (e.g. diaphyseal vs. proximal) or time to mobilization after femur fracture and only accounts for the presence or absence of a femur fracture. Further, it is well known that BMI may not be the best at representing obesity in adolescents and body fat measurements [44,45]. The absence of psychosocial factors such as socioeconomic status, family support, and access to healthcare also limits the comprehensiveness of the analysis. The TQIP dataset lacks data on the timing and protocols for mobilization and rehabilitation post-fracture, which are important for understanding recovery trajectories and outcomes. However, the size of the study population provided by the database strengthens this study. In addition, this study was able to distinguish the associated risk between specific lower extremity fractures.

## Conclusions

OAs involved in MVCs have a graded higher associated risk of severe lower extremity fractures, with the femur being the only bone at higher risk. OAs also have a longer LOS and require additional support upon discharge, including home-health services or inpatient rehabilitation. Future research is needed to confirm the higher risk of femur fractures in OAs due to MVCs and to determine the potential biomechanical or physiological causes behind this increased risk. Emphasizing early discharge planning with comprehensive inpatient and outpatient rehabilitation or earlier mobilization, if warranted, could help shorten LOS or reduce the need for additional services upon discharge.

## Funding sources

This research did not receive any specific grant from funding agencies in the public, commercial, or not-for-profit sectors.

## Ethics approval

Ethics approval was not required as this was a deidentified database study.

## CRediT authorship contribution statement

**Shaelyn Choi:** Writing – original draft, Methodology, Formal analysis, Data curation, Conceptualization. **Jeffry Nahmias:** Writing – review & editing, Methodology, Formal analysis, Data curation, Conceptualization. **Matthew Dolich:** Writing – review & editing, Methodology, Conceptualization. **Michael Lekawa:** Writing – review & editing, Methodology, Conceptualization. **Brian R. Smith:** Writing – review & editing, Methodology, Conceptualization. **Ninh Nguyen:** Writing – review & editing, Methodology, Conceptualization. **Areg Grigorian:** Writing – review & editing, Supervision, Project administration, Methodology, Formal analysis, Data curation, Conceptualization.

## Declaration of competing interest

The authors have no conflict of interests to declare that are relevant to the content of this article.
